# Schizophreniform disorder. Clinical manifestations and diagnosis. Purposely a case

**DOI:** 10.1192/j.eurpsy.2024.1542

**Published:** 2024-08-27

**Authors:** S. M. Bañón González, N. Ogando Portilla, B. Gamo Bravo, M. E. González Laynez, N. Sekade Gutiérrez, F. García Sánchez

**Affiliations:** ^1^Hospital Universitario Infanta Sofía, Madrid; ^2^Hospital Universitario de Toledo, Toledo; ^3^Hospital Universitario de Móstoles, Madrid, Spain

## Abstract

**Introduction:**

Schizophreniform Disorder is described pretty similar to schizophrenia, but with the difference of the symptoms duration which have to last for at least 1 month but less than 6 months. Patients have to be back at their baseline functional level once the disorder has resolved. This is a heterogeneous group of patients who have either a disorder similar to schizophrenia or something closer to a mood disorder.

**Objectives:**

To analyze clinical, psychopathological and epidemiological characteristics of schizophreniform disorder and also review causes, incidence, prevalence, diagnostic, therapeutic tools and the importance of maintaining the treatment, because of the abandonment of the treatment, which is a predictor of relapses.

**Methods:**

A review of the main impact literature concerning schizophreniform disorder is done during the last five years: prevalence, incidence, pathogenesis and its relationship with other psychiatric disorders encoded in DSM-V are studied.

**Results:**

The etiology is unknown. Psychotic symptoms can be treated with antipsychotics for 3 to 6 months. They usually respond faster than patients with schizophrenia (75% vs 20% respond within 8 days).

**Conclusions:**

The disease has a favorable prognosis, and has similarities with mood disorders. However, some data suggest a close relationship to schizophrenia. In support of the relationship with mood disorders, patients have more affective symptoms and a better outcome than patients with schizophrenia.

**Disclosure of Interest:**

None Declared

